# Characterization of Sleep Structure and Autonomic Dysfunction in REM Sleep Behavior Disorder

**DOI:** 10.1109/OJEMB.2024.3397550

**Published:** 2024-05-07

**Authors:** Nicla Mandas, Maximiliano Mollura, Giulia Baldazzi, Parisa Sattar, Maria Mura, Elisa Casaglia, Michela Figorilli, Laura Giorgetti, Pietro Mattioli, Francesco Calizzano, Francesco Famà, Dario Arnaldi, Monica Puligheddu, Danilo Pani, Riccardo Barbieri

**Affiliations:** Hadron AcademyIstituto Universitario di Studi Superiori, IUSS 534441 27100 Pavia Italy; Department of Electrical and Electronic EngineeringUniversity of Cagliari 3111 09124 Cagliari Italy; Department of Electronic, Information and BioengineeringPolitecnico di Milano 18981 20156 Milano Italy; Department of Electrical and Electronic EngineeringUniversity of Cagliari 3111 09124 Cagliari Italy; Interdepartmental Sleep Disorder Research CenterUniversity of Cagliari 3111 09124 Cagliari Italy; Department of Electrical and Electronic EngineeringUniversity of Cagliari 3111 09124 Cagliari Italy; Department of Medical Sciences and Public Health, Sleep Disorder Research CenterUniversity of Cagliari 3111 09124 Cagliari Italy; Department of Electrical and Electronic EngineeringUniversity of Cagliari 3111 09124 Cagliari Italy; Department of Medical Sciences and Public Health, Sleep Disorder Research CenterUniversity of Cagliari 3111 09124 Cagliari Italy; Department of Neuroscience (DINOGMI)University of Genoa 9302 16126 Genoa Italy; Department of Neuroscience (DINOGMI)University of Genoa 9302 16126 Genoa Italy; Neurophysiology unitIRCCS Ospedale Policlinico San Martino 9246 16132 Genoa Italy

**Keywords:** Autonomous nervous system, heart rate variability (HRV), Markov chains, point process, REM sleep behavior disorder

## Abstract

*Goal:* REM Sleep Behavior Disorder (RBD) is a REM parasomnia that is associated to high risk of developing α-synucleinopathies, as Parkinson's disease (PD) or dementia with Lewy bodies, over time. This study aims at investigating the presence of autonomic dysfunctions in RBD subjects, with and without PD, by assessing their sleep structure and autonomous nervous system activity along the different sleep stages. *Methods:* To this aim, an innovative framework combining a sleep transition model, by Markov chains, with an instantaneous assessment of autonomic state dynamics by statistical modeling of heart rate variability (HRV) dynamics through a point-process approach, was introduced. *Results:* In general, RBD groups showed lower HRV than controls across all sleep stages, as well as higher probabilities of transitioning towards lighter sleep stages. Subjects also affected by PD present an even lower HRV, but better sleep continuity. *Conclusions:* RBD patients suffer from sleep fragmentation and overall autonomic dysfunction, mainly due to lower autonomic activation across all sleep stages. Coexistence of PD seems to improve sleep quality, possibly due to a sleep-related relief of their symptoms.

## I. Introduction

Sleep, which is defined as a transitory state during which we are not able to actively interact with the surrounding environment [1], plays a key role in people wellbeing. Its macrostructure can be divided into rapid eye movements (REM) phase and further three sleep stages (N1, N2, and N3) of non-REM (NREM) sleep [2]. In absence of any sleep disorder, N1 acts as a transition between sleep and wake (W) states, paving the way for N2 and, finally, N3, as sleep deepens. Lastly, in the last and more active phase of the sleep cycle, i.e., the REM phase, bursts of rapid eye movements occur, as well as distinct temporal patterns and brain-wave interactions [3]. Differently from NREM stages, REM presents more sudden variations in the measurable physiological parameters, such as abrupt changes in arterial blood pressure, heart rate (HR), respiration, and a drop in body temperature. In this stage, healthy subjects generally show complete muscle atonia, i.e., a type of muscular immobility caused by the total relaxation of the muscles [2].

This study focuses on a characteristic parasomnia of the REM sleep stage called REM Sleep Behavior Disorder (RBD). People affected by this disease lose the normal muscle atonia of REM stage and engage in dream-enactment behaviors [4]. When it is not associated with other neurodegenerative disorders, RBD is defined as isolated or idiopathic (iRBD). RBD patients often develop other peculiar α-synucleinopathies like Parkinson's disease (PD, 41.9%) [5], dementia with Lewy bodies (50.5%), and multiple system atrophy (7.5%) [6]. Specifically, RBD can be a prodromic non-motor symptom of PD. Although the incidence of iRBD only reaches 1% in the general population older than 60 years, about 90% of them receive a diagnosis for α-synucleinopathy within their 15-year follow-up, with PD representing 45% of the cases [7]. Currently, PD is the most common disease of the central nervous system after Alzheimer. Its main feature is the presence of Lewy bodies, aggregates of the α-synuclein protein that form at the level of the pars compacta of the substantia nigra, one of the five basal ganglia [8].

This study aims at characterizing the presence of autonomic dysfunctions during different sleep stages in patients affected by iRBD, and in patients affected by both RBD and PD (PD-RBD). Results were statistically compared together with a control group of unaffected people. This objective was pursued by evaluating the transition probabilities between different sleep stages and by performing statistical comparisons to assess the presence of any significant difference among the three populations. To associate these differences with possible autonomic dysfunction, heart rate variability (HRV) indexes have been estimated by statistical modeling of the cardiovascular system during the first sleep cycle.

The proposed analysis framework innovatively combines a sleep transition model (including the characterization of sleep macrostructure) with an instantaneous assessment of autonomic state dynamics in a causal way. Such integration allows for the identification of biomarkers of the concomitant evolution of both sleep and HRV parameters in the populations of interest. As a result, the sleep and autonomic features highlighted in this study can both characterize RBD in its idiopathic and PD-converted forms, and eventually add peculiar signatures to allow for their prediction or diagnosis.

## II. Materials and Methods

Participants were enrolled between 2013 and 2021 by the Italian Sleep Medicine Centers of Cagliari and Genova (Italy), both accredited by the Italian Association of Sleep Medicine. The study was conducted in accordance with the Declaration of Helsinki and was approved by local Ethics Committee of the University Hospital of Cagliari (PG/2018/11699) and IRB of University Neurology Clinics at Policlinico San Martino in Genoa (IRB approval n. 105/2023 – DB id 13027), and patients gave their consent for their data to be anonymously processed. In particular, this study was carried out on a retrospective collection of clinical and PSG data. Participants with iRBD or PD-RBD were diagnosed following the diagnostic criteria of the International Classification of Sleep Disorders – third edition (ICSD-3) and the Movement Disorder Society Clinical Diagnostic Criteria for PD. The Cagliari's dataset consisted of 41 polysomnographic (PSG) recordings performed at the Interdepartmental Centre for Sleep Medicine of the University of Cagliari with the Morpheus EEG and PSG Holter (Micromed S.p.A., Treviso, Italy). The Genova's dataset consisted of 37 PSG recordings performed at the Sleep and epilepsy center of the IRCCS San Martino Hospital of the University of Genova with the BE Plus LTM and MizAR 40 (EBNEuro S.p.A., Firenze, Italy). From an engineering perspective, the different equipments of the two laboratories were comparable and did not introduce any bias in the analysis. For both centers, sleep scoring was performed manually in 30-second epochs, according to the American Academy of Sleep Medicine scoring criteria [9] whose procedure is also included in the last guidelines for video-PSG-based diagnosis of RBD from the International RBD Study Group [10].

Inclusion criteria in common across all three populations were: the presence of an electrocardiographic (ECG) signal of acceptable quality in each sleep stage (regardless of its duration) during the first sleep cycle; absence of arrhythmias, cardiac pacing, sleep apneas, or sleep-related breathing disorders according to ICSD-3; absence of pharmacological treatments that alter HR or HRV. While for the affected groups no other neurological or sleep diseases other than RBD and PD were admitted, for the inclusion of participants into the control group, the absence of any neurological or sleep disease was required. Furthermore, since levodopa medications are proven to affect EEG synchronization [11], sleep quality [12], and HRV, and in particular it might be associated with less or more disruptive self-regulatory processes, in ON and OFF states respectively, as observed in PD patients exhibiting motor symptoms like freeze-of-gait [13], in this study all recruited PD-RBD patients were naïve from dopaminergic therapy. Based on these premises, the study population consisted of a cohort of 27 patients with iRBD (age 67 ± 7, 63% males), 27 patients with PD-RBD (age 68 ± 8, 74% males), and 24 unaffected people forming a control group (CG, age 60 ± 10, 46% males). Furthermore, their Apnea-Hypopnea Index (AHI) values were 2.96 ± 3.07, 3.85 ± 5.34, 5.01 ± 4.18 events/h, whereas their Periodic Limb Movement Index (PLMI) values were 12.67 ± 17.11, 13.29 ± 15.38, 12.78 ± 15.81 events/h, for the CG, iRBD, and PD-RBD groups respectively.

### A. Sleep Structure Analysis

To delineate the sleep structure from the hypnograms, Markov chains were employed to model the transition probabilities of passing from one stage to another. While continuous-time Markov chains were successfully used to characterize sleep structure [14], in this work we considered a time-homogeneous Markov chain, as the one described in [15], to be applied to full-night hypnograms previously synchronized on the sleep onset, up to their last sleep stage different from wake. Given that all participants had at least five hours of sleep, we limited the analysis to five hours of data for each patient.

A discrete Markov chain $\{ {{{X}_t}} \}$ is defined as a stochastic process satisfying the following criteria [16]: the state space $S$ is a finite and countable set, in our case corresponding to $S = \{ {W,\ N1,N2,N3,REM} \}$; the time index set is defined as $T = \{ {0,\ 1,\ 2, \ldots } \}$; and the conditional transition probability only depends on the current state:

\begin{align*}
&\Pr \left\{ {{{X}_{n + 1}} = j{\mathrm{|}}{{X}_0} = {{i}_0}, \ldots,{{X}_{n - 1}} = {{i}_{n - 1}},{{X}_n} = i} \right\} \\
&\qquad\qquad\quad = \Pr \left\{ {{{X}_{n + 1}} = j|{{X}_n} = i} \right\} \tag{1}
\end{align*}

for all $n \in T$ and all states ${{i}_0}, \ldots,{{i}_{n - 1}},i,j$. Markov chains require the transition probabilities to be constant. One way to overcome this limit is to test whether the transition probabilities reported in the matrices are time-independent, as in [17]. Instead, we opted for dividing the 5-hour hypnograms into non-overlapping segments to pursue stationarity in the transition probabilities. The length of such segments was fixed to 60 minutes, after a preliminary analysis to find such optimal value (data not shown), and discrete Markov chains [7], [8] were computed in each one. Transition probabilities ${{p}_{ij}}$ from state $i$ to state $j$ of Markov chain $\{ {{{X}_t}} \}$ can be computed as

\begin{equation*}
{{p}_{ij}} = \frac{{{{n}_{i \to j}}}}{{\mathop \sum \nolimits_j {{n}_{i \to j}}}} \tag{2}
\end{equation*}

where ${{n}_{i \to j}}$ represents the number of transitions from state $i$ to state $j$, while the denominator represents the total number of transitions starting from state $i$. In this way, the overall probability to jump from state $i$ to all the other possible states sums to one. For our sleep macrostructure analysis, these probabilities are ordered in a $5 \times 5$ matrix:

\begin{equation*}
\left[ {\begin{array}{ccccc} {{{p}_{W \to W}}}&{{{p}_{W \to N1}}}&{{{p}_{W \to N2}}}&{{{p}_{W \to N3}}}&{{{p}_{W \to REM}}}\\ {{{p}_{N1 \to W}}}&{{{p}_{N1 \to N1}}}&{{{p}_{N1 \to N2}}}&{{{p}_{N1 \to N3}}}&{{{p}_{N1 \to REM}}}\\ {{{p}_{N2 \to W}}}&{{{p}_{N2 \to N1}}}&{{{p}_{N2 \to N2}}}&{{{p}_{N2 \to N3}}}&{{{p}_{N2 \to REM}}}\\ {{{p}_{N3 \to W}}}&{{{p}_{N3 \to N1}}}&{{{p}_{N3 \to N2}}}&{{{p}_{N3 \to N3}}}&{{{p}_{N3 \to REM}}}\\ {{{p}_{REM \to W}}}&{{{p}_{REM \to N1}}}&{{{p}_{REM \to N2}}}&{{{p}_{REM \to N3}}}&{{{p}_{REM \to REM}}} \end{array}} \right] \tag{3}
\end{equation*}

Once the normality of distributions was rejected by the Lilliefors test, transition probabilities were tested by the unpaired Mann-Whitney U test, followed by the correction via the false discovery method (FDR) [18], to investigate the presence of any pairwise difference in sleep continuity between groups. Test significance was set at *p*-value < 0.05.

### B. Cardiovascular Characterization

To assess autonomic changes during sleep, ECG signals were extracted from PSG recordings and up-sampled, when needed, to the highest available sampling frequency in the dataset (i.e., 512 Hz), to provide a homogeneous temporal resolution for the identification of the R-peaks. For each participant, ECG waveforms were analyzed with a custom Pan-Tompkins-based QRS-detection software [19], which extracted the sampling instants associated with the R-peak events in the signal. The resulting tachograms were automatically analyzed and then manually reviewed to correct for missing beats and extrasystoles. During this phase, extra beats originated by ectopic sources other than the sinoatrial node, such as premature ventricular contractions, were excluded from the analysis, since misleading with respect to the underlying activity of the autonomic nervous system (ANS). Statistical modeling of the resulting inter-beat interval series was then computed and used to estimate HRV indexes.

The time-varying nature of the proposed modeling approach allows to pause the statistical estimation in the presence of irregular RR segments and resume it slightly after, thus avoiding the generation of erroneous frequency contents due to non-sinus rhythms.

Standard, window-based HRV methods have been already used in this field [20], [21], [22], [23] but they present some limitations due to the uneven sampling of the tachogram, which usually requires resampling by interpolating functions, thus overlooking the intrinsic point process nature of the RR interval series, and to the lack of a goodness-of-fit metric when parametric approaches (e.g., autoregressive estimates) are considered. Importantly, none of the existing methods provide an instantaneous estimate of HR and RR interval variance, thus requiring the analysis of long signal segments, typically longer than five minutes, which is cumbersome in this application, as some sleep stages could exhibit a shorter duration and faster dynamics.

To overcome these limitations, we applied a model that exploits the stochastic structure of the heartbeat intervals by modeling it as a history-dependent inverse Gaussian (HDIG) process as shown in [24]. The HDIG model is defined as:

\begin{align*}
f\left( {t{\mathrm{|}}{{H}_{{{u}_k}}},\theta } \right) =& {{\left[ {\frac{{{{\theta }_{p + 1}}}}{{2\pi {{{\left( {t - {{u}_k}} \right)}}^3}}}} \right]}^{\frac{1}{2}}}\\
&\times\exp \left\{ { - \frac{1}{2}\frac{{{{\theta }_{p + 1}}{{{\left[ {t - {{u}_k} - \mu \left( {{{H}_{{{u}_k}}},\theta } \right)} \right]}}^2}}}{{\mu {{{\left( {{{H}_{{{u}_k}}},\theta } \right)}}^2}\left( {t - {{u}_k}} \right)}}} \right\} \tag{4}
\end{align*}

where ${{H}_{{{u}_k}}}$ is the history of previous RR intervals up to the R-peak ${{u}_k}$, and the average RR interval $\mu $ is obtained as a regression over the past $p$ RR intervals (being $p$ the order of the autoregressive model). The shape parameter of the inverse Gaussian (${{\theta }_{p + 1}}$) and its variance (${{\sigma }^2}$), along with $\mu $, can vary over time. A local maximum likelihood method is used to estimate the set of unknown parameters $\theta ( t )$.

The use of this probabilistic framework allows for the instantaneous estimation of the average RR interval ($\mu $), internal variance ($\sigma $), and of the indexes derived from the spectral analysis. In sleep studies, the advantages of applying a point-process approach consist in providing continuous estimates for the HRV indexes, thus accounting for the strong non-stationarity of HRV oscillations during sleep. Continuous estimates for these time-varying parameters allowed us to not restrain our analysis to segments longer than 5 minutes. Based on a preliminary analysis (data not shown), we empirically set the order to $p$=9, and the temporal resolution to 0.05 s.

In the frequency-domain, it is possible to identify the very low frequency (VLF, 0.01–0.04 Hz), low frequency (LF, 0.04–0.15 Hz), and high frequency (HF, 0.15–0.4 Hz) components. Physiological interpretation of the VLF component is still under scrutiny [25], even though in healthy adults it seems to reflect long-term autonomic control related with circadian rhythms and hormonal changes, among others [26]. The LF component reflects the activity of both sympathetic (SNS) and parasympathetic (PNS) nervous system activity as modulated by the baroreflex, a homeostatic mechanism that helps us balancing the arterial blood pressure values. The HF component is associated with the faster modulation of PNS [27], mainly affected by respiration through Respiratory Sinus Arrhythmia [28]. The ratio between the LF and the HF component is known as the sympathovagal balance, although the interpretation of this value must always consider the context in which the signal was acquired and the health status of the subject [29].

For each sleep stage (i.e., N1, N2, N3, and REM) in the first sleep cycle, we considered only the first occurrence, and computed the following parameters: RR interval (${{\mu }_{RR}}$), variance ($\sigma _{RR}^2$), power in the LF band ($L{{F}_{RR}}$), power in the HF band ($H{{F}_{RR}}$), normalized LF power ($LF{{n}_{RR}}$), normalized HF power ($HF{{n}_{RR}}$), and ratio between the power in the two bands ($LF/H{{F}_{RR}}$). The normalized values were obtained by dividing the corresponding power band by the difference between the total power and the power in the VLF band, for every time instant.

For each parameter and sleep stage, continuous estimates of HRV indexes have been averaged for statistical analyses. The confidence bounds for such parameters can be seen in Table I. After rejecting the normality of the distributions by using the Lilliefors test, the non-parametric Kruskal-Wallis test was used to assess the presence of significance among all populations, while between-group differences were assessed by unpaired Mann-Whitney U test and corrected via the FDR method. For this analysis, we studied both the absolute parameter values and the differences between REM and NREM values (Δ) for each parameter. Conversely, within-group differences were evaluated by the paired Mann-Whitney U test between all the possible combinations of REM and NREM sleep stages. Again, adjusted p-values were considered.

**TABLE I:** HRV Parameters

Group	Sleep stage	${{\mu }_{RR}}$ [msec]	$\sigma _{RR}^2$ [msec^2^]	$L{{F}_{RR}}$ [msec^2^]	$H{{F}_{RR}}$ [msec^2^]	$LF{{n}_{RR}}$ [-]	$HF{{n}_{RR}}$ [-]	$LF/H{{F}_{RR}}$ [-]
CG	N1	928.±164	492±179	345±267	162±74	0.593±0.213	0.346±0.173	6.9±3.3
	N2	943±175	445±190	261±105	156±78	0.561±0.269	0.373±0.241	3.5±1.9
	N3	950±134	410±169	296±150	176±92	0.517±0.215	0.422±0.187	2.5±1.6
	REM	932±97	481±211	425±287	130±45	0.691±0.205	0.253±0.196	7.5±5.3
iRBD	N1	952±146	151±82	65±25	63±36	0.444±0.224	0.504±0.162	1.6±0.7
	N2	962±141	162±121	93±85	71±45	0.482±0.160	0.466±0.146	2.5±0.8
	N3	977±177	208±114	92±68	97±71	0.489±0.230	0.465±0.225	2.3±0.9
	REM	961±144	203±126	121±93	58±34	0.600±0.231	0.338±0.187	5.0±1.6
PD-RBD	N1	934±141	94±58	53±35	42±27	0.464±0.227	0.480±0.260	2.8±1.3
	N2	941±154	110±63	57±34	50±33	0.504±0.230	0.446±0.219	2.1±1.2
	N3	956±104	193±101	125±50	76±59	0.473±0.231	0.474±0.316	2.3±0.7
	REM	945±104	291±102	166±61	111±25	0.584±0.206	0.354±0.177	3.0±1.7

The table shows the confidence bounds for each HRV parameter computed by the point process, according to the sleep stage and group, expressed as mean value ± the standard deviation.

Finally, to merge information coming from sleep structure and ECG-derived features and correct for confounding effects, generalized linear models have been developed. Indeed, we employed regression models to predict the outcome of a subject given the value of an HRV feature in a specific sleep stage, while correcting for confounding effects such as their age, sex, and the center where the PSG took place. Conversely, linear mixed-effects models were used to see if the information regarding the population and sleep stages was significant in characterizing the feature value.

Analyses of both sleep structure and HRV have been carried out in MATLAB v2022a.

## III. Results

### A. Sleep Structure

The analysis between the CG and the iRBD group is the only one presenting statistically significant transitions after correcting the p-values by the FDR method, specifically during the third hour of sleep. Overall, the iRBD group moves more easily towards lighter sleep stages: in the transition from stage N2 to wake, the probability was higher for the iRBD group (i.e., ${{p}_{N2 \to W}}$ = 0.054) than for the CG (${{p}_{N2 \to W}}$ = 0.029), despite statistical significance being lost when FDR correction is considered. At the same time, probabilities of maintaining a sleep stage were lower for the iRBD (e.g., during the third hour of sleep, ${{p}_{N3 \to N3}}$ = 0.963 for the CG, and 0.936 for the iRBD group, with *p* < 0.05).

Comparing PD-RBD with CG, the former has also lower self-transitions for wake (${{p}_{W \to W}}$ = 0.724, versus 0.800), N3 (${{p}_{N3 \to N3}}$ = 0.932, versus 0.956), and REM (${{p}_{REM \to REM}}$ = 0.879, versus 0.955), even though no statistical significance was obtained after FDR correction.

Regardless of statistical significance, when it comes to the two RBD groups, the PD-RBD patients present higher probabilities of maintaining a sleep stage (e.g., N2 both during the second and the fourth hour of sleep). Conversely, iRBD patients showed a tendency of moving towards lighter sleep stages (e.g., ${{p}_{N1 \to W}}$ = 0.164 for the iRBD group during the third hour of sleep, and 0.061 for the PD-RBD group), and maintaining the wake, as suggested by their higher probability in the third hour of sleep (${{p}_{W \to W}}$ = 0.847, 0.724 for the PD-RBD group).

The resulting significant sleep transitions after FDR correction are reported in Table II.

**TABLE II:** Significant Sleep Transitions

Hour of sleep	From	To	Probability for CG	Probability for iRBD	*p* - value
3^rd^	W	W	0.800	0.847	0.002
3^rd^	N1	W	0.068	0.164	0.005
3^rd^	N2	REM	0.023	0.010	0.038
3^rd^	N3	N3	0.963	0.936	0.031
3^rd^	REM	REM	0.955	0.920	0.038
4^th^	REM	REM	0.940	0.906	0.036

The table shows the statistically significant sleep transition differences during the 5-hour sleep, with their respective probability and resulting corrected *p*-value, in the three pairwise comparisons.

### B. Cardiovascular Characterization: Between-Group Analysis

Among the developed generalized linear models, we have verified that the simplest one, which considers the covariates but excludes the interactions between them, is able to yield the same results as the more complex models, while maintaining a lower computational cost and assuring easier interpretability. For this reason, such model and the related findings are described hereinafter, while the remaining models and their results are reported in the supplementary material, Section II-B-E.

The following logistic regression model was implemented, for each sleep stage separately:

\begin{equation*}
\text{logit}\left( \mathrm{y} \right)\ \sim 1 + \mathrm{X} + \mathrm{A} + \mathrm{S} + \mathrm{C} \tag{5}
\end{equation*}

where $\mathrm{y}$ represents the outcome (i.e., the population), $\mathrm{X}$ the HRV feature value, $\mathrm{A}$ the age of the subject, $\mathrm{S}$ the sex of the participant (Male: 0, Female: 1), $\mathrm{C}$ the center (i.e., Cagliari or Genova, respectively encoded as 0 and 1).

When considering the CG and the iRBD groups, most of the models returned *p* < 0.05 associated with age, which is justified by the fact that iRBD people in our dataset are, on average, 7 years older than the controls. For $L{{F}_{RR}}$, feature value $\mathrm{X}$ was statistically significant (*p* < 0.05) during N1, N2, and REM, with negative estimates of the feature's coefficient, i.e., a decrease in the value of $\mathrm{X}$ translates into a higher probability of being an iRBD participant. During N1, we also have significant results for the parameters $LF{{n}_{RR}}$ (with a negative estimate of the feature's coefficient, so an increase in its value points toward a higher probability of belonging to the CG) and $HF{{n}_{RR}}$ (with a positive estimate of the coefficient, so an increase in its value gives a higher probability of being an iRBD participant).

By testing the models between the CG and the PD-RBD group, we obtained significant results during N1 and N2 for $\sigma _{RR}^2$, $L{{F}_{RR}}$, $H{{F}_{RR}}$, all with negative estimates of the corresponding coefficient. This means that we have a lower probability of being a PD-RBD participant when their values increase. $L{{F}_{RR}}$ is also significant during REM, always with a negative estimate for $\mathrm{X}$.

We did not obtain significant results when considering the two RBD groups: the only significant coefficient in most of the cases was the center.

To assess the potential impact of other covariates, the AHI and the PLMI have been added to the regression model. Interestingly, in such analyses, age and sex lose their significance in several parameters when comparing CG and affected populations, and a similar trend is seen for the center covariate in RBD populations, thus strengthening our findings.

The obtained HRV results are comparable with those that can be obtained by a standard statistical approach such as the unpaired Mann-Whitney U test followed by the FDR correction, which can be found in the supplementary material, Section II-A.

The statistical analysis performed on the Δ values didn't provide any statistically significant result. However, the interested reader may refer to the supplementary material, Section II-F for these findings.

### C. Cardiovascular Characterization: Within-Group Analysis

Within-group differences were found only when considering the REM phase and the NREM sleep stages.

**Fig. 1.**
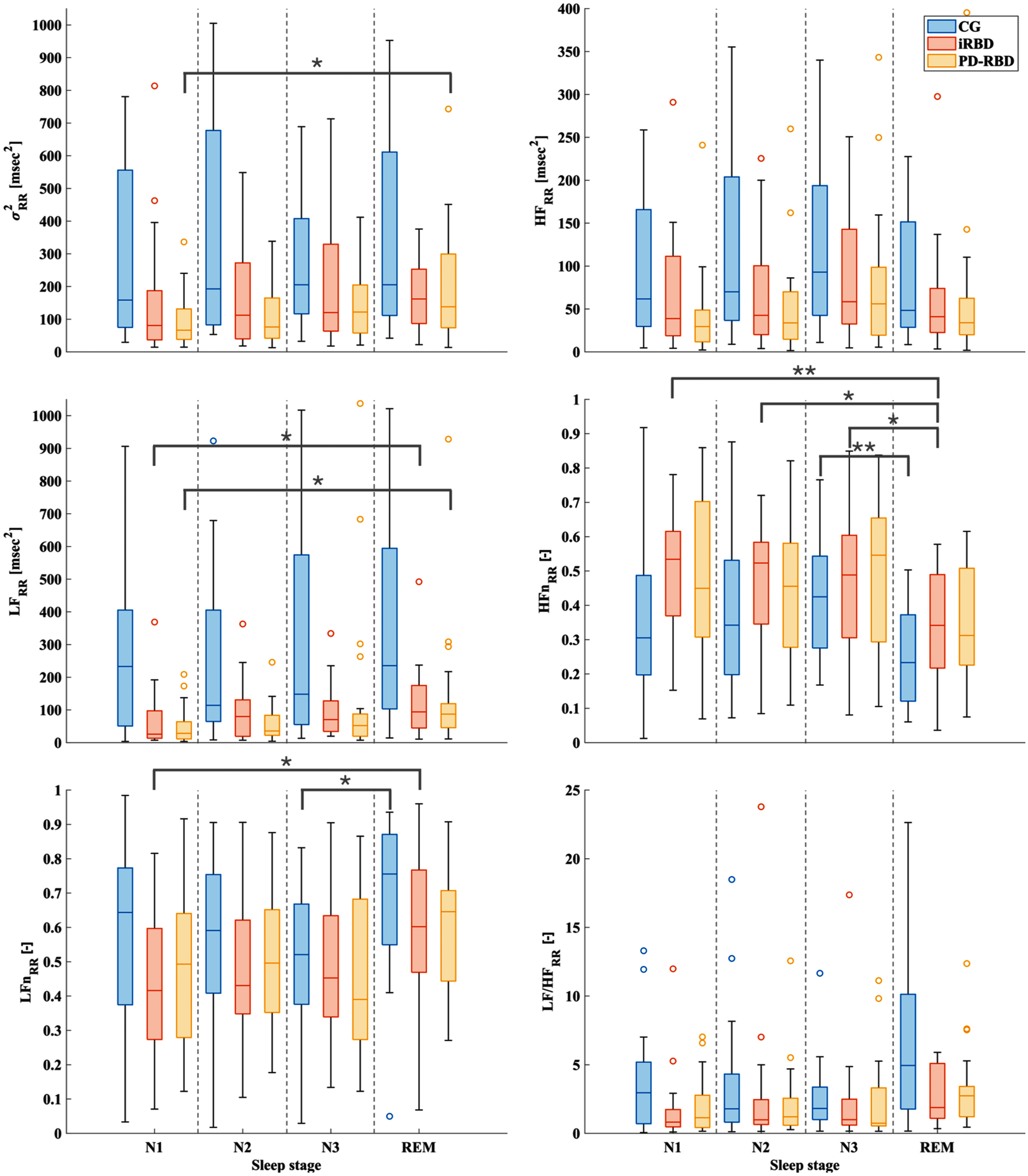
Results for variance $\sigma _{RR}^2$, power in the low frequency band $L{{F}_{RR}}$, normalized low frequency $LF{{n}_{RR}}$, power in the high frequency band $H{{F}_{RR}}$, normalized high frequency $HF{{n}_{RR}}$, and sympathovagal balance $LF/H{{F}_{RR}}$ for the three populations analyzed (CG, iRBD, PD-RBD) across the four sleep stages. For the sake of clarity, results are reported in restricted ranges. For $\sigma _{RR}^2$, the range is [0, 1050], outliers up to 3380 msec^2^ are not depicted. For $L{{F}_{RR}}$, the range is [0, 1050], outliers up to 2060 msec^2^ are not shown. For $H{{F}_{RR}}$, the range is [0, 400] and the outliers up to 980 msec^2^ are not reported. For $LF/H{{F}_{RR}}$, the range is [0, 25] and the outliers up to 82 are not depicted. $LF{{n}_{RR}}$, $HF{{n}_{RR}}$, and the $LF/H{{F}_{RR}}$ are unit-less due to their computations. Significance for the within-group analysis is represented as follows: ^*^:p<0.05; ^**^:p<0.01. In each box, the median of the distribution is represented by the central line, while the 25^th^ and the 75^th^ percentiles are the bottom and top edges of the box, respectively. Extreme values that are still considered to fall into the distribution are represented by the whiskers, while outliers are indicated by the circles.

For the CG, $LF{{n}_{RR}}$ and $HF{{n}_{RR}}$, were significantly different between REM sleep stage and N3 (*p* < 0.05 and *p* < 0.01, respectively). Specifically, $LF{{n}_{RR}}$ showed an increase of median value from REM to N3, and $HF{{n}_{RR}}$ showed instead a decrease of median value from N3 to REM, as it can be seen from Fig. 1.

In the iRBD group, significant differences are found between the REM and the N1 sleep stages for the parameters $L{{F}_{RR}}$ and $LF{{n}_{RR}}$, which showed an increase from N1 to REM (*p* < 0.05), and $HF{{n}_{RR}}$, which conversely exhibited a decrease from N1 to REM (*p* < 0.01). For $HF{{n}_{RR}}$, also N2 and N3 differ significantly from the REM sleep stage with the same trend (*p* < 0.05).

For the PD-RBD participants, $\sigma _{RR}^2$ and $L{{F}_{RR}}$ significantly differed between N1 and REM sleep stages (*p* < 0.04), with an increased median value during the REM sleep stage.

## IV. Discussion

Sleep analysis highlighted how both RBD groups, when compared with controls, showed a more fragmented sleep. This is revealed by statistically significant lower probabilities of maintaining a given sleep stage and higher probabilities of transitioning towards lighter sleep stages. This is in line with previous studies conducted by Christensen et al. [30], whose results point to a less stable REM sleep phase in both iRBD and PD-RBD subjects, with respect to controls. Also, even if the CG subjects show a more stable sleep when compared to the pathological groups, it has been previously demonstrated that they still exhibit a significant degree of asymmetry in sleep-stage transitions, meaning that the number of transitions going from stage 1 to stage 2 doesn't match the number of transitions going in the other way. This characteristic asymmetry of the healthy subjects gets lost with sleep disorders [31].

Furthermore, between the two RBD groups enrolled in our study, participants affected also by PD (i.e., PD-RBD) suffered from less fragmented sleep than iRBD ones, with a lower tendency to maintain the wake stage. This aspect should be carefully considered in light of the manual sleep staging that has been performed in this study. Indeed, even though several automatic data-driven sleep scoring algorithms have been developed and could better emphasize sleep fragmentation in PD [30], further studies are needed to validate these tools on large subject cohorts and to integrate them into scoring recommendations. For this reason, only manual sleep staging is nowadays clinically recognized.

In this regard, there is still no definite explanation of the interaction between sleep and motor functions for the PD-RBD group. As some patients report an improvement in motor function upon awakening, thanks to the release of dopamine [32], the alleviation of these symptoms could be the reason why they manage to have less fragmented sleep.

Results of the analysis of HRV parameters showed reduced variability in the RBD groups, with lower median values across all sleep stages with respect to the controls. In the iRBD group, $L{{F}_{RR}}$ values were lower during the REM stage than N1 and N2. Both iRBD and PD-RBD groups presented a reduction in $H{{F}_{RR}}$ when passing from N3 to REM, but not as strong as the one reported for the control group, suggesting a general reduction in the variability in their autonomic regulation. By looking at the median values for the sympathovagal balance, we can notice how the range was narrow for the iRBD group (going from 0.877 during N2 to 1.112 during REM), while a higher variability was evident for the PD-RBD group (range 0.631 ÷ 2.051), and an even higher one in the CG (range 1.362 ÷ 3.670), supporting the hypothesis that an overall lower variability characterizes RBD groups.

These findings are not in contrast with the scientific literature. A study by Sorensen et al. demonstrated that the mean RR interval was not significantly different among the three analyzed populations [33], as in our analyses. In [20], Bugalho et al. demonstrated the presence of a lower variance across all sleep stages in subjects affected by RBD with respect to controls. They also demonstrated how $H{{F}_{RR}}$ tends to decrease during REM sleep stage in the CG while keeping a value closer to the one during NREM sleep in the iRBD group [34].

Given these previous findings, it can be speculated that the missing LF variability activation in the RBD groups might be due to a shorter duration of the considered sleep stage, indicating that the impaired ANS of these subjects is not able to follow the ideal changes in such a fragmented and rapidly evolving sleep.

To the best of our knowledge, the present study is the first to model the statistical properties of the inter-beat interval series of people affected by RBD and PD and relate the resulting instantaneous HRV indexes with the sleep structure. In this regard, the developed models may help in providing an evolving sequence of snapshots of the dynamic evolution of sleep and the autonomic system in CG, iRBD, and PD-RBD populations, due to the precise match allowed between hypnograms and HRV estimates, thus resulting in a probability of population outcome. The main innovation of the adopted methodology is that it allows to monitor time-varying changes in the physiological state of the subject, focusing on sleep stage-by-stage transitions of ANS control throughout the night, overcoming typical standard window-based HRV analysis limits.

Further studies will explore the obtained results on the cardiovascular characterization (particularly focusing on blood pressure control) during the entire night of sleep, to have a comprehensive interpretation of the HRV parameters change throughout the night given a specific sleep stage. This assessment, along with the Markov-based transition model introduced in this work, might support the prediction of sleep scoring. More data is also needed to validate the generalized linear models, with the ultimate goal of developing a predictive model for the early detection of RBD and its possible phenoconversion into PD-RBD. Remarkably, even though we considered only the first sleep cycle and an offline analysis, our sleep and autonomic features can provide even more accurate assessments when exploited in full-night PSG, and eventually prove to be considered as novel digital biomarkers: indeed, such data-driven models may lead to a significant shift in RBD diagnosis and management in online monitoring and for the introduction of new technologies for sleep study in the operating room and the intensive care unit.

## V. Conclusion

We developed a methodology to investigate the presence of statistically significant differences in both nocturnal sleep continuity and autonomic regulation, between patients affected by RBD and patients affected by both RBD and PD, with respect to control subjects.

This study demonstrates that RBD patients suffer from both sleep fragmentation and overall autonomic dysfunction, mainly due to a lower SNS activation during the REM sleep stage, which translates into lower values for the power in the low-frequency band. Overall, RBD patients also present a lower variability across all sleep stages with respect to unaffected controls.

Sleep structure is altered in the RBD groups due to the lower probabilities of keeping a specified sleep stage, but when comparing the two groups it was noticeable how PD-RBD patients exhibit a more regular sleep, possibly due to a sleep-related relief of their symptoms.

In conclusion, as it is possible to observe autonomic dysfunction prior to the occurrence of the first symptoms, this study holds a significant clinical relevance. An accurate assessment of autonomic state changes in specific sleep transitions could be exploited to compute a marker for early diagnosis of RBD, or PD, potentially resulting in a better quality of life for the affected patients.

## Supplementary Materials

The readers are encouraged to read the Supplementary Materials to have a more comprehensive view of the developed generalized linear models for this study, as described in the Methods section, and of the obtained results, as well as additional statistical tests that were performed.

Supplementary materials

## Conflicts of Interest

The authors declare that they have no conflict of interest.

## Author Contributions

N. M., Maximiliano M., G. B., D. P., and R. B. contributed to the conception and design of the study. E. C., M. F., L. G., P. M., F. C., F. F., D.A., M. P. contributed to the data collection. N. M., Maximiliano M., Maria M., and R. B. contributed to the code writing. N. M., Maximiliano M., G. B., P. S., Maria M., D. P., and R. B. contributed to the data analysis. N. M., Maximiliano M., G. B., P. S., Maria M., M. F., D. A., M. P., D. P., and R. B. contributed to the data interpretation. N. M., Maximiliano M., and G. B. contributed to drafting the manuscript.

All authors read and approved the submitted manuscript.
