# Supplementary material for: Characterization of Sleep Structure and Autonomic Dysfunction in REM Sleep Behavior Disorder
Source: IEEE Open J Eng Med Biol. 2024 May 7;5:859–66. doi: 10.1109/OJEMB.2024.3397550 (PMC11573400; doi:10.1109/OJEMB.2024.3397550)
Supplement: Supplementary materials [file supp1-3397550.pdf]

Supplementary Materials

Characterization of Sleep Structure and Autonomic Dysfunction in REM Sleep Behavior Disorder

Nicla Mandas, *Student Member*, *IEEE*, Maximiliano Mollura, *Member*, *IEEE*, Giulia Baldazzi, *Member*, *IEEE*, Parisa Sattar, *Student Member*, *IEEE*, Maria Mura, Elisa Casaglia, Michela Figorilli, Laura Giorgetti, Pietro Mattioli, Francesco Calizzano, Francesco Famà, Dario Arnaldi, Monica Puligheddu, Danilo Pani, *Senior Member*, *IEEE*, and Riccardo Barbieri, *Senior Member*, *IEEE*

In this work, generalized linear models have been developed to merge the information about sleep structure with ECG-derived features associated with the autonomic nervous system activity. They fall into two main categories: (I) a set of logistic regression models (*A, B, C*) were developed to predict the outcome of a subject given the value of an HRV feature in a specific sleep stage while correcting for confounding effects; (II) a set of linear mixed-effects models (*D*) were used to correlate feature values with information about the population and on the sleep stage. The following labels for the outcome were used when testing the populations pairwise: CG 0 – iRBD 1, CG 0 – PD-RBD 1, iRBD 0 – PD-RBD 1. For the linear mixed-effects models the labels were CG 0, iRBD 1, PD-RBD 2.

I. Methods

A. 1st logistic regression model

In the simplest case, the implemented model considered the feature value and covariates such as the age of the subject, the sex of the participant, and the center where the PSG took place. Details concerning this first model can be found in section B of the Results paragraph in the main manuscript.

B. 2nd logistic regression model

In the second model, information about the sleep stage was introduced in the formula with respect to the first one.

To do so, three indicator variables were added to encode the four possible sleep stages (N1, N2, N3, and REM), represented by S1, S2, and S3, with N1 stage as reference. The formula was:

$$\mathrm{logit}(y) \sim 1 + X + A + S + C + S1 + S2 + S3 \tag{2}$$

In this way, a single model for each parameter was obtained by considering all sleep stages at the same time.

C. 3rd logistic regression model

A possible interaction between the feature value X and the indicator variables encoding the different sleep stages was also investigated, with respect to the second model presented. Therefore, in a third model, also the last wake before sleep onset was included, specifically the last 30 seconds. To do so, another indicator variable (S4) was introduced. In this case the reference is the wake phase. The formula was:

$$\mathrm{logit}(y) \sim 1 + X + A + S + C + S1 + S2 + S3 + S4 \\ + X * S1 + X * S2 + X * S3 + X * S4 \tag{3}$$

D. Linear Mixed-Effects Models

In this model, the three populations together have been tested. The outcome in this case was represented by the feature value itself. The formula was the following:

$$y \sim ID + PD + A + S + C + S1 + S2 + S3 + S4 \\ ID * S1 + ID * S2 + ID * S3 + ID * S4 \\ PD * S1 + PD * S2 + PD * S3 + PD * S4 + 1|SUBJECT \tag{4}$$

where ID and PD represent indicator variables encoding the population (if zero, they refer to a participant of the CG), while S1, S2, S3, and S4 are indicator variables enclosing the four sleep stages and the last wake before sleep onset, which represents the reference model.

E. Other covariates

The addition of clinical information such as the AHI and PLMI indexes was tested for all the presented models, in the form of covariates.

F. NREM and REM differences

Furthermore, differences between NREM and REM sleep stages were tested to highlight any potential mismatch between the studied populations. Specifically, for each given HRV parameter, the difference between its value during the REM sleep stage and its value during each NREM sleep stage was computed and tested between the three populations. Accordingly, the difference between the feature value in REM and the feature value in N1 is represented as Δ1, the same goes for N2 (Δ2) and N3 (Δ3).

II. Results

A. 1st logistic regression model and HRV statistics

Results of this model can be found in the main manuscript (Results, Section B).

In any case, they are in line with those obtained by applying the unpaired Mann-Whitney U test. For the latter, RR interval series μ_{RR} did not show a statistically significant difference between groups in any analyzed sleep stage.

In N1 and N2, a significant difference was found between the CG and the PD-RBD group for σ^2_{RR} ($p = 0.018$ for both). Median σ^2_{RR} values were higher in the CG in all sleep stages when compared with the affected populations, while among them iRBD patients presented higher median variance than the PD-RBDs, as it can be seen from Fig. 1 in the main manuscript.

The power in the LF band, LF_{RR}, was lower in all sleep stages in both RBD groups when compared with the CG, with values

TABLE I
BETWEEN-GROUP STATISTICAL ANALYSIS

Group 1	Group 2	N1	N2	N3	REM
CG	iRBD	LF_{RR}*			LF_{RR}*
		HFn_{RR}*			
CG	PD-RBD	σ^2_{RR}*	σ^2_{RR}*		
		LF_{RR}**	LF_{RR}**	LF_{RR}*	LF_{RR}*
		HF_{RR}*	HF_{RR}*		

The table shows the HRV parameters that are statistically significant after the FDR correction in the between-group analysis. Significance is indicated as follows: *:p< 0.05; **p<0.005.

even lower for the PD-RBD patients. However, statistical significance was found across all sleep stages for the comparison between CG and PD-RBD ($p < 0.005$ in N1 and N2, and $p < 0.05$ in N3 and REM), and for N1 and REM between CG and iRBD ($p < 0.05$).

When comparing the CG and the PD-RBD, HF_{RR} significantly differentiated the two populations during N1 and N2, with lower values for the PD-RBD ($p = 0.029$ and $p = 0.030$, respectively). Instead, its normalized value was significantly different between CG and iRBD during N1, with a lower median value for CG ($p = 0.047$).

A summary of the significant HRV parameters between the analyzed population is reported in Table I.

B. 2nd logistic regression model

By keeping fixed the sleep stage, it can be noticed how the increase in σ^2_{RR}, LF_{RR}, HF_{RR}, and LFn_{RR}, is associated with a reduced probability of being an iRBD participant when compared with the CG.

Instead, testing the CG with the PD-RBD group resulted in a decreased probability of being a PD-RBD participant as the values of the following parameters increase: μ_{RR}, σ^2_{RR}, LF_{RR}, HF_{RR}.

The mean RR interval μ_{RR} returned a $p < 0.05$ when testing the iRBD group against the PD-RBD group, with a negative estimate of the feature's coefficient, suggesting that a higher RR interval characterizes an iRBD participant when keeping fixed the sleep stage.

These findings are in line with the ones obtained with the previously described models, highlighting the fact that more complex models to describe this dynamic are not strictly needed.

C. 3rd logistic regression model

LF_{RR} was significant when testing CG and iRBD groups, along with the coefficients for age and sex, but no interaction exhibited a p-value close to the significance threshold, thus leading to the same result of the less complex models.

The same goes for the variance and the power in both the LF and HF bands when evaluating the PD-RBD group against the CG.

Results about the two RBD groups were not significant, apart from the variance σ^2_{RR} which returned a $p = 0.075$, close to the significance threshold. In this case, the interaction between the feature value and the REM sleep stage was significant ($p < 0.05$), meaning that the behavior of the variance is significantly different between the REM sleep stage and the reference model, i.e., the last wake before sleep onset.

D. Linear Mixed-Effects Models

Looking at the resulting model for σ^2_{RR}, both ID and PD showed $p < 0.05$, pointing out a significantly different variance between the controls (reference model) and the two RBD populations. For both, the estimate was negative, resulting in a lower variance in iRBD and PD-RBD groups than CG.

For LF_{RR} and HF_{RR}, PD was significant with a negative estimate, so PD-RBD participants had lower power in the corresponding frequency bands with respect to their CG counterparts.

For the power in the LF band, S4 differed significantly during REM with respect to the reference model ($p < 0.005$).

The sympathovagal balance varied significantly between the CG and the iRBD ($p < 0.001$) for ID, with a negative estimate: therefore, we can infer that a lower value is associated with the diseased population. All the features' coefficients encoding the sleep stages were significantly less than zero with $p < 0.001$, so they all differed significantly from the reference model. This result suggests that a lower sympathovagal balance in all sleep stages is associated with the presence of the disease always with respect to the last wake before sleep onset.

E. Other covariates

By adding the information provided by the AHI and PLMI indexes to our models, results do not change significantly. In some cases, the variables A, S, and C do not result to be significant anymore ($p > 0.05$), while in most cases the AHI is significantly different between the populations. Specifically, the AHI coefficient has a positive estimate across all models, meaning that a higher value characterizes an RBD participant (both iRBD and PD-RBD) when evaluating the model with the

control group, while it characterizes a PD-RBD subject in the comparison with an iRBD one.

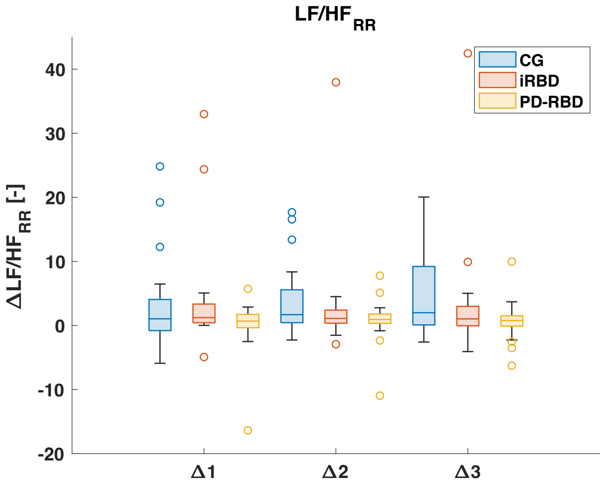

Fig. 1. Results for the sympathovagal balance LF/HF_{RR} for the three populations analyzed (CG, iRBD, PD-RBD) across the three Δ values considered. For the sake of clarity, results are reported in restricted ranges, specifically in the range [-20,45], not showing an outlier in Δ1 for the CG group at -60.

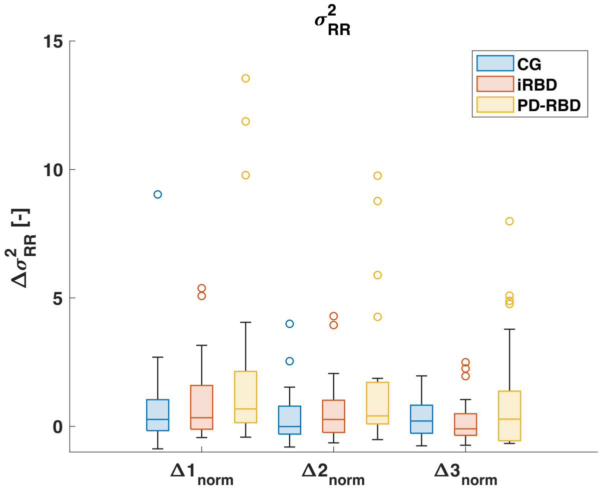

Fig. 2. Results for the variance σ^2_{RR} for the three populations analyzed (CG, iRBD, PD-RBD) across the three Δ values considered, in the normalized scenario. For the sake of clarity, results are reported in restricted ranges, specifically in the range [-1,15], not showing an outlier in $\Delta 1_{norm}$ for the iRBD group at around 30, and an outlier in $\Delta 2_{norm}$ for the iRBD group at around 25.

F. NREM and REM differences

No parameter variation resulted significantly different between the groups after FDR correction. Still, by looking at the boxplots presented in Figure 1, it can be noted how the median difference in the sympathovagal balance between REM and N1 is bigger for the iRBD group than the PD-RBD group (p-value = 0.036 before correction), a trend that can be seen for all NREM sleep stages.

Comparing the CG with the PD-RBD subjects, the median difference in LF/HF_{RR} between REM and N2 is bigger for the CG instead of the PD-RBD group (see Figure 1), confirming a higher variability in the CG group across different sleep stages.

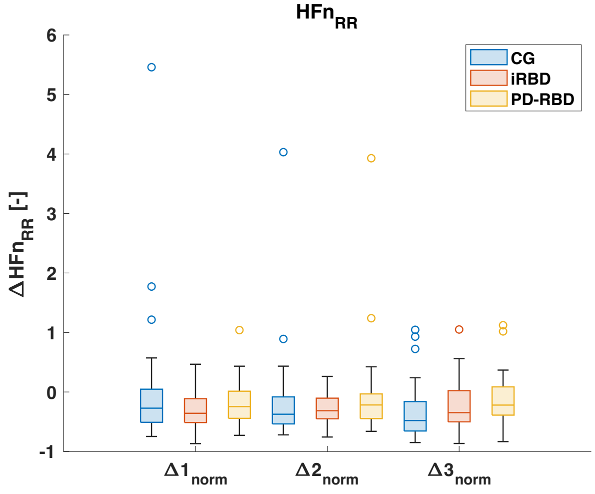

Fig. 3. Results for the normalized HF power HFn_{RR} for the three populations analyzed (CG, iRBD, PD-RBD) across the three Δ values considered, in the normalized scenario.

Another test that has been performed was normalizing these differences by the value of the feature during the corresponding NREM sleep stage (e.g., for $\Delta 1_{norm}$ the difference of the feature value between REM and N1 has been consequently divided by the value during N1). In this scenario, the variance had a lower median difference in the CG group compared to the PD-RBD group (p-value = 0.039 before correction) between REM and N2 (see Fig. 2). Considering instead the N3 sleep stage, we see the same behavior with the parameter HFn_{RR} (see Fig. 3).

G. Summary of the developed GLM

The findings obtained with the generalized linear models were in line with those obtained with the other performed statistical analysis. Indeed, keeping fixed the sleep stage, an increase in σ^2_{RR}, LF_{RR}, HF_{RR}, and LFn_{RR}, was associated with a reduced probability of being an iRBD participant when compared with the CG. Conversely, when testing the PD-RBD group with the CG, the probability of being a PD-RBD participant decreased as the value of μ_{RR}, σ^2_{RR}, LF_{RR}, HF_{RR} parameters increased.

Another highlight of this analysis was that more complex models are not necessarily needed to characterize the studied populations. Hence, the first logistic regression model was chosen given its low computational cost, yielding in any case the same results as the more complex models, also assuring easier interpretability.
